# The Association between Replacement Drivers and Depressive Symptoms

**DOI:** 10.3390/ijerph20010575

**Published:** 2022-12-29

**Authors:** Jongmin Lee, Heejoo Park, Juyeon Oh, Juho Sim, Chorom Lee, Yangwook Kim, Byungyoon Yun, Jin-Ha Yoon

**Affiliations:** 1 Department of Occupational Health, Graduate School of Public Health, Yonsei University, Seoul 03722, Republic of Korea; 2 Department of Business Administration and Data Science, CHA University, 120 Haeryong-ro, Donggyo-dong, Pocheon-si 11160, Republic of Korea; 3 Department of Public Health, Graduate School, Yonsei University, Seoul 03722, Republic of Korea; 4 Department of Preventive Medicine, Yonsei University College of Medicine, Seoul 03722, Republic of Korea; 5 The Institute for Occupational Health, Yonsei University College of Medicine, Seoul 03722, Republic of Korea

**Keywords:** replacement driver, gig worker, depressive symptoms, paid workers, machine learning

## Abstract

A replacement driver is a type of gig worker who provides driving services to the target point with the drunk driver’s own car. This study aimed to examine the association of replacement drivers (ref: paid workers) with depressive symptoms. Information on replacement drivers was collected through online/offline surveys. Data from the 8th Korea National Health and Nutrition Examination Survey were applied to construct the control group. The Patient Health Questionnaire-9; ≥5 points was defined as depressive symptoms. Adjusted odds ratios (aOR) and 95% confidence intervals (CI) were calculated by performing multivariable logistic regression analysis. The mean age of replacement drivers was 56.11. The prevalence of depressive symptoms in replacement drivers and controls were 49.63% and 12.64%, respectively. Replacement drivers showed a higher association with depressive symptoms than paid workers (aOR 7.89, 95% CI [5.53–11.26]). This relationship was prominent in the older, low-education, and low-income groups. Linear discriminant analysis was the most effective in predicting depressive symptoms among the machine learning models. Using the replacement driver feature increased the AUC values of the models. Given the strong association between depressive symptoms and replacement drivers, in-depth studies to establish guidelines to prevent mental diseases among replacement drivers are required.

## 1. Introduction

A gig worker, also known as a platform laborer, refers to an independent worker who is not employed by any company and could be characterized as being on-demand, online platform-based, and temporary. Owing to the development of new technologies and related information, the number of digital labor platforms and gig economy workers has rapidly increased worldwide in the past decade [1]. Gig workers in the US were estimated to be 34% of the entire workforce, which was approximately 55 million workers in 2017, and it was predicted to be 43% in 2020 owing to the coronavirus disease 2019 (COVID-19) outbreak [2]. In South Korea, the number of gig workers is estimated at about 2.2 million, which is 8.5% of the working population, according to a report from the Korea Employment Information Service in 2021. Although the number of gig workers has increased, studies on their health issues is lacking.

Owing to the limited studies and extraordinary characteristics of gig workers, several controversies have arisen regarding the pros and cons of this online platform-mediated work, especially regarding health outcomes [3,4]. Some advocates insist that the opportunity and flexibility of the work might result in net benefits on health outcomes [5], whereas other opponents have highlighted the precarity and working environment as related to poorer health outcomes [6,7,8]. These characteristics of gig workers may differ based on the traits of the task for each individual. In-depth research on the health outcomes of gig workers based on the characteristics of their work should be conducted. In particular, considering precarious employment and its association with mental health [9], studies examining the mental health of gig workers should be prioritized among various health outcomes.

One of the most common types of gig economy workers is delivery workers. There are two types of delivery workers: food or manufactured goods delivery and human delivery services. Replacement drivers, also called substitute or *Daeri* drivers, are online platforms that mediate human delivery using customer cars in South Korea and provide driving services to drunk people with their own cars [10]. Owing to heavy alcohol consumption and frequent field sobriety tests in Korea, the demand for replacement drivers has been considerable [11]. These drivers are specifically exposed to frequent night shift work, emotional stress from dealing with drunk customers, and a tense environment while driving customers’ cars, which could result in aggravation of health conditions, including mental disease, chronic disease, and injury [12,13]. Hence, research on the health outcomes of gig economy workers is needed.

The job duties of replacement drivers include occupational health issues related to emotional labor, night shift work, and driving. There has been considerable research on the association between depression and emotional labor in service workers. However, these studies focused on workers in systematized workplaces; therefore, these results might not be generalizable to gig economy workers. A previous study conducted by Jung et al. presented the significant association between workplace violence and depression among replacement drivers, who tend to frequently experience several types of violence [10]. Nonetheless, studies comparing depressive symptoms between replacement drivers and other representative populations are lacking. Furthermore, an independent association between gig economy workers and depression beyond night shift characteristics is still lacking.

Hence, this study aims to determine the psychological health issues of gig economy workers. To examine this association, we analyzed replacement driver data and compared it with the general population. Furthermore, job-specific characteristics were discussed in light of our current results regarding depressive symptoms.

## 2. Materials and Methods

### 2.1. Survey Data for Replacement Drivers

**Figure 1:**
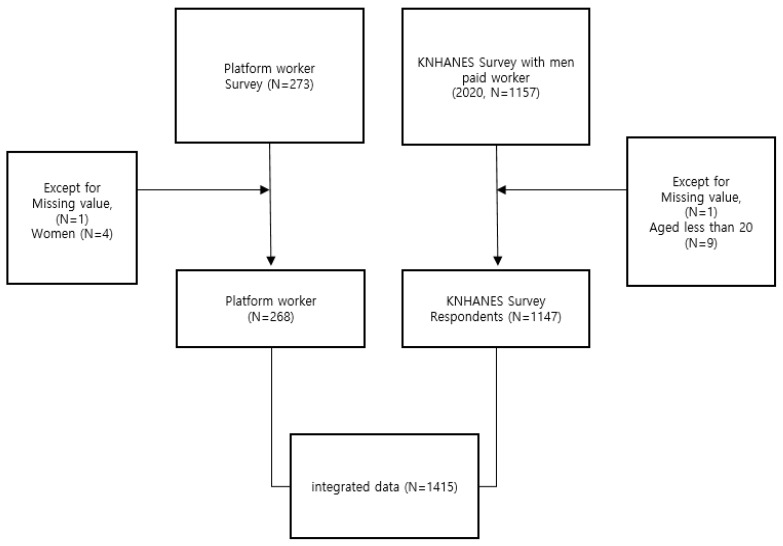
Participant enrollment process.

Survey data of gig workers from the replacement driver’s branch of the Federation of Korean Trade Unions, which has 1,308,000 subscribers (as of December 2021), were collected online and offline to investigate the demographic and occupational characteristics and psychological and chronic disease status of replacement drivers. The survey was conducted from November 2021 to September 2022. All participants provided informed consent prior to participating in the survey. From a total of 273 participants responded to the survey, participants with missing value (N = 1) and women (N = 4) were excluded owing to the small sample size. A total of 268 participants remained in the study (Figure 1).

### 2.2. Korea National Health and Nutrition Examination Survey Data

The data from the Korea National Health and Nutrition Examination Survey (KNHANES), whose participants were used as a control group, were based on samples extracted using stratified colony sampling methods based on demographic criteria such as residential area, gender, and age. The KNHANES is a credible and representative cross-sectional nationwide survey on the health, nutritional, and health awareness status of Koreans. This study used the 8th KNHANES data published in 2020, and through this, the data were processed and used as secondary data. From a total of 1157 male paid workers, 1147 participants remained in the analysis after excluding those aged less than 20 years (*n* = 9) or those with missing variables (*n* = 1).

### 2.3. Main Variables (Replacement Driver Group)

Gig workers are ultra-short-term temporary workers who make short-term or temporary contracts for specific tasks on digital platforms and receive remuneration. A replacement driver is a gig worker who drives someone else’s car to their destination under a short-term contract. In this study, the two groups were compared and analyzed by selecting ordinary paid workers with earned income from the data of replacement drivers directly recruited by the Korea Agency Driving Cooperative and the Korea National Health and Nutrition Examination Survey data to determine the difference between gig and general workers during the COVID-19 pandemic.

### 2.4. Primary Outcome (PHQ-9 Questionnaire)

In this study, the Patient Health Questionnaire (PHQ-9) scale, which has been validated in Korea, was used as a screening tool to determine depressive symptoms. The PHQ-9 is a self-reported questionnaire developed by Spitzer et al. in 1999 [14] that can diagnose major depressive disorders. The PHQ-9 consists of a 4-point Likert scale, and each item is selected according to the frequency of symptoms: “not at all” at 0 points, “several days” at 1 point, “more than half days” at 2 points, and “nearly every day” at 3 points. The criteria for determining symptoms of depression are usually “no symptoms” for less than 5 points, “light depressive symptoms” for 5–9 points, and “depression” for more than 10 points. In this study, it was judged that there were “depressive symptoms” when the total score of each item of the PHQ-9 was more than five points, according to a general standard, and “severe depressive symptoms” when it was more than 10 points.

### 2.5. Covariates

In the questionnaire surveying replacement drivers, the variables used in the study consisted of the same questions as those of the KNHANES. Participants were classified into replacement drivers and the general population, according to the data source. Age, education level, household income level, working hours, smoking status, alcohol consumption history, days of muscular exercise, and sleeping time were included as covariates. The smoking status of the study participants was stratified into three groups. Those who have never smoked in their lives are “none,” those who used to smoke but do not now are “ex-smokers,” and those who smoke now are “current smokers.” The frequency of alcohol consumption among participants was stratified into three groups: “none” for those without alcohol consumption; “social drinking” for those who drink alcohol 1–4 times a month; and “heavy” for those who drank more than once a week. Education level was divided into “below high school” and “above university.” Income levels were defined as “high” for household income of more than 40 million KRW per year and “low” for household income of less than 40 million KRW per year. Working hours were defined as “short” and “long” for less and more than 40 h per week, respectively. Muscular exercise from 0 to 1 and more than 1 days were defined as “none” and “exerciser” group, respectively. Sleeping time from 5 to 10 h was defined as “normal” group, and sleeping time for less than 5 h per day or more than 10 h per day was defined as the “abnormal” group.

### 2.6. Statistical Analyses

First, the difference in baseline characteristics stratified by depressive symptoms of replacement drivers were examined by performing the chi-square and Student’s *t*-test for categorical and continuous variables, respectively. A multivariate logistic regression model was used to calculate the odds ratio (OR) and 95% confidence interval (CI). Age, education level, household income level, working hours, smoking status, alcohol consumption history, days of muscular exercise, and sleeping time were used as covariates. Poisson regression was performed as a sensitivity analysis. We investigated the association between depressive and severe depressive symptoms using the variable between replacement drivers and general workers. Moreover, we investigated the association of depressive symptoms with permanent position workers, temporary position workers, and replacement drivers.

A subgroup analysis was performed to identify factors that may contribute to heterogeneity. The variables used included age, educational level, and household income level. Age was divided into “old” group (50 years or older) and “young” group (under 50 years). Educational level was divided into two groups: participants with a bachelor’s or higher education level (above university) and those with a lower education level (below high school). Household income level was also divided into two groups: “high” for household income more than 40 million KRW per year and “low” for household income less than 40 million KRW per year.

We used propensity score matching (PSM) to reduce the confounding bias between participants. We used the nearest neighbor method with a ratio of 1:1 and a caliper width of 0.2. The variables used for PSM were age, educational level, household income level, working hours, smoking status, alcohol consumption history, muscular exercise days, and sleeping time. The baseline characteristics and estimation of adjusted ORs of depressive symptoms between replacement drivers and paid workers were examined using matched data.

Machine learning was performed to predict depressive symptoms using various features including age, educational level, household income level, working hours, smoking status, alcohol consumption history, muscular exercise days, and sleeping time. The proportion of the training set was 70%. All continuous features were normalized using Z-score. The training set was oversampled to adjust the imbalanced nature of the data. Generalized Linear Model (GLM), Linear Discriminant Analysis (LDA), Gradient Boosting Algorithm (GBM), and Quadratic Discriminant Analysis (QDA) were employed to predict depressive symptoms. In addition, deep learning technique was also performed using Keras with Tensorflow. The balanced accuracy and area under the curve (AUC) value from the receiver operating characteristic (ROC) curve was estimated.

A *p*-value of <0.05 was considered statistically significant for all two-sided statistical tests. All data analyses were performed using R software (version 4.1.1, http://cran.r-project.org/ (accessed on 1 November 2022)). Machine learning was performed using the package “Caret.” Python (version 3.10.6) was used for deep learning model implementation.

## 3. Results

Of the 1415 participants, the average age (standard deviation) of the participants is 48.09 (14.04) years (46.21 [14.45] for paid workers vs. 56.11 [8.25] for replacement drivers; *p* < 0.001). The number of paid workers was 1147 (81.06%). The total prevalence of depressive symptoms was 19.65% (*n* = 278); 52.16% (*n* = 145) for general paid workers and 47.84% (*n* = 133) for replacement drivers. Table 1 shows the characteristics of the study population according to the presence or absence of a replacement driver. A total of 912 people (64.45%) had university-level education or higher, and their average household income was 52.04 million won. The replacement drivers had a significantly higher proportion of older age, low income, current smoking, no alcohol consumption, and abnormal sleeping time than paid workers (all *p* < 0.01). There were no significant differences in education level and working hours. Table S1 shows the differences in participants’ baseline characteristics stratified by replacement driver and type of work position. Replacement drivers also had a significantly higher proportion of all variables, except muscular exercise, than permanent or temporary position workers (all *p* < 0.001). Table S2 shows the multivariable logistic regression model of depressive symptoms among work positions. The difference between temporary (ref: permanent position worker, adjusted OR = 1.11, 95% CI = 0.76–1.61) and permanent position workers was slight, whereas the difference in OR values between platform workers (adjusted OR = 8.27, 95% CI = 5.56–12.30) and temporary position workers was large.

Table 2 shows the characteristics of the study population according to the presence or absence of depressive symptoms. The replacement drivers had a significantly higher proportion of depressive symptoms. The depressive symptoms group had a significantly higher proportion of low income, current smokers, non-muscular exercise, and abnormal sleeping time (all *p* < 0.01). Moreover, there were no significant differences in age, education level, alcohol consumption, or working hours.

According to the logistic regression models, the crude OR (95% CI) of depressive symptoms in replacement driver was 6.80 (5.06–9.15). Furthermore, the age-adjusted ORs (95% CI) of depressive symptoms in the replacement driver group are 9.09 (6.49–12.74) compared with those of the general paid workers. Replacement drivers indicated a higher adjusted OR (95% CI) of depressive symptoms (8.59, 6.10–12.09) than the general workers after adjustment for age and socioeconomic status in the multivariable logistic regression model (Table 3). Moreover, models with further adjustments for age, education, household income, smoking, alcohol consumption, sleep hours, working hours, and muscular exercise also showed a significant relationship between depressive symptoms and replacement drivers (ref: paid worker, adjusted OR = 7.89, 95% CI = 5.53–11.26). The analysis with Poisson regression also reproduced the significant association of depressive symptoms with replacement drivers (Table S3). The association between severe depressive symptoms (PHQ-9 ≥ 10) and replacement drivers was also similar (Table S4).

**Figure 2:**
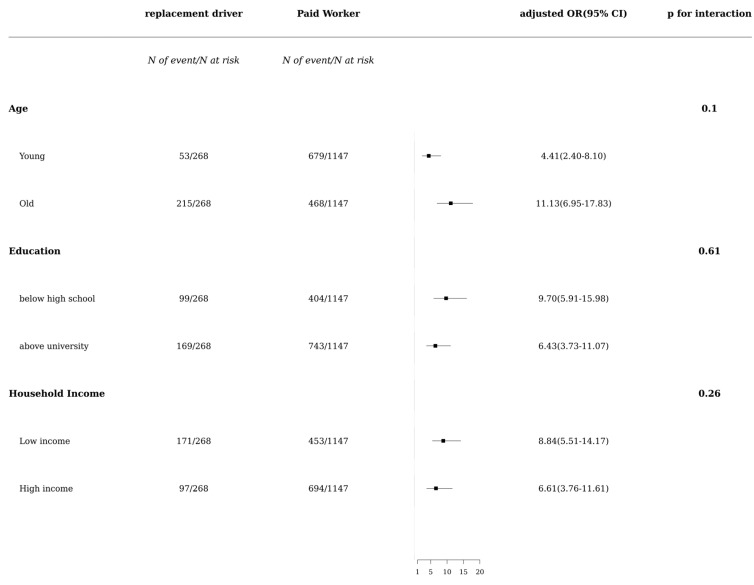
Forest plot of subgroup analysis.

According to the subgroup analysis by age, education, and income level, there was a more prominent association of depressive symptoms with replacement drivers (ref: paid workers) in old age, below high school, and low-income groups (adjusted OR: 11.13 [6.95–17.83] for old age, adjusted OR: 9.70 [5.91–15.98] for below high school, adjusted OR: 9.70 [5.91–15.98] for low income, all *p* for interaction >0.05; Figure 2).

Moreover, there was no significant difference in any of the covariates between paid workers and replacement drivers after PSM (all *p* > 0.05). Depressive symptoms were significantly higher in replacement drivers than in paid workers (*p* < 0.001, Table S5). After PSM, we found that replacement driver was a statistically significant predictor of depressive symptoms (OR: 7.86 [4.99–12.39]).

**Figure 3:**
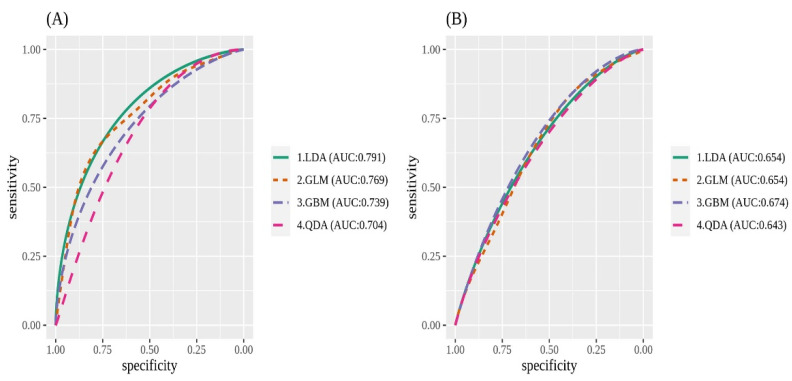
Differences in the area under the curve (AUC) based on whether (**A**) replacement driver variable was used or (**B**) not.

**Figure 4:**
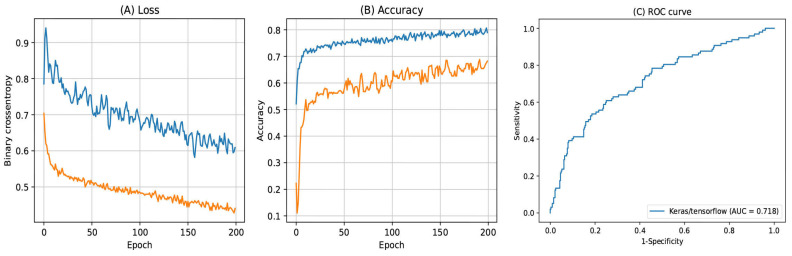
(**A**) Loss, (**B**) Accuracy, and (**C**) ROC curve of the deep learning model with Tensorflow.

The balanced accuracy and AUC value of each machine learning model are summarized in Table S6 and Figure 3. Among them, LDA presented the best performance (balanced accuracy 0.719, AUC 0.791). When comparing the models with or without the replacement driver variable, the balanced accuracy and AUC values were considerably higher in the model with the replacement driver variable. In the deep learning model with Tensorflow, the accuracy and AUC values were 0.795 and 0.718, respectively (Figure 4).

## 4. Discussion

This study examined the association between depressive symptoms and replacement drivers (ref: paid workers). This relationship was maintained even after adjusting for demographic, socioeconomic, and lifestyle covariates. Moreover, in the subgroup analysis, old age, low education, and low-income groups presented a prominent association of depressive symptoms with replacement drivers compared with paid workers. Sensitivity analyses, including those considering the work position of paid workers, PSM, and a higher cut-off of depressive symptoms (PHQ-9 ≥ 10), also reproduced similar results. Replacement drivers had a significantly higher prevalence of abnormal sleep hygiene and a lower socioeconomic status than paid workers. Moreover, according to machine learning analysis, LDA was the most effective in predicting depressive symptoms. Using replacement driver feature considerably increased the accuracy and AUC values of the models.

The significant association of depressive symptoms with replacement drivers, compared with the general population, might be explained by several factors. First, Bajwa et al. classified the health and social vulnerabilities of gig workers into three categories: occupational vulnerabilities, precarity, and platform-based vulnerabilities [15]. Occupational vulnerability depends on the work performed, such as musculoskeletal problems owing to routines or traffic accidents while driving. Replacement drivers are highly vulnerable to traffic accidents because their insurance for driving others’ cars may not completely cover unexpected tragedies. Precarity, which could exist in other businesses or occupations, aligns with the contingent nature of platform-mediated tasks [16]. Platform-based vulnerabilities, unlike occupational vulnerabilities or precarity, are specifically related to gig workers in terms of several issues, including worker misclassification, social isolation, and pricing control. Gig workers using online platforms, usually misclassified as self-employed, cannot negotiate the price of their work and easily experience racial and gender discrimination [16]. Moreover, although online rating systems are a fundamental component of the world constructed by online platforms, they are a critical factor in determining workers’ stress. In the case of Uber drivers, racial and gender stereotypes might considerably affect the rate, despite being late for other reasons, including bad traffic or an accident [17]. Platform laborers might be related to mental health concerns owing to platform-specific vulnerabilities as well as existing occupational vulnerabilities and precarity.

In addition to the aforementioned vulnerabilities, replacement drivers in South Korea have several other factors that may be significantly associated with depressive symptoms. Replacement drivers usually work at night and might be susceptible to depression based on the results of previous studies demonstrating that night shifts are significantly associated with an increased risk of depression [18]. The association between these could be explained by several plausible mechanisms, including effects on the promoter methylation of the serotonin transporter gene (SLC6A4) [19] and circadian disruption of glucocorticoid oscillations [20] from the environmental stress of night shifts.

Another factor is related to the driver’s occupation. Despite scarce research, few previous studies have examined the significant association between drivers, notably truck drivers, and depressive symptoms [21,22,23]. According to these studies, truck drivers with irregular working schedules reported poor sleep quality, expressed as short sleep duration and excessive daytime sleepiness [24,25]. Furthermore, cab drivers presented a higher prevalence of poor mental status owing to unhealthy lifestyle habits, including physical inactivity and junk food consumption [26] as well as common exposure to poor and hazardous working conditions, such as irregular shift work, long working hours, and environmental pollution [27,28]. Similarly, our study presented unhealthy lifestyle factors, which might imply a significant association between depression and replacement drivers.

Another vulnerability to depressive symptoms among replacement drivers is socioeconomic status. Replacement drivers are older, earn less, and have a lower social status than paid workers. Socio-economic status is a major determinant of health in later life. In particular, household income affects mental health more than education level [29]. A replacement driver usually operates at an undetermined time and location. Consequently, replacement drivers have more inconsistent income and economic weaknesses than general paid workers. According to Helena [30], short-term workers have poorer mental health and higher job instability than full-time workers. Therefore, job instability and income anxiety among replacement drivers who work part-time, with schedules that are not fixed and harm their mental health, may be associated with depressive symptoms.

To the best of our knowledge, this is the first study to analyze the psychological health of replacement drivers in detail. Considering the extreme scarcity of research on gig economy workers, further studies should be conducted on this population. Another strength of this study is the relatively large research population, which consisted of general paid workers and replacement drivers.

Our study had several limitations. First, it was a cross-sectional study that could not clarify the causal relationship between replacement drivers and depressive symptoms. This implies that there is a possibility for replacement drivers to have depressive symptoms at the first time. Considering the precarity and vulnerability of replacement drivers, individuals who were already in a difficult situation could start working as replacement drivers. Thus, future studies with well-designed cohorts should be conducted to examine causal relationships. Second, critical factor information for determining depressive symptoms may be lacking. To accurately determine the symptoms of depression, a psychiatric history is key, such as depression and medication history; however, it does not exist. Thus, there were unmeasured confounders, which could have resulted in a bias. In the same context, there was lack of information about participants’ career path or type of personality, which is associated with psychological characteristics. We attempted to minimize bias by correcting for depressive symptom questionnaire, socioeconomic status, and lifestyle directly related to depressive symptoms, nonetheless, further studies should address these limitations with detailed information. Third, while conducting the survey, errors may have occurred because of a high dependence on past memories. However, an objective questionnaire called the PHQ-9 was used, and errors were minimized by making questions as detailed as possible.

## 5. Conclusions

In conclusion, our study highlighted a significant association between depressive symptoms and replacement drivers and paid workers. Considering the weakness of replacement drivers, including abnormal sleep hours and low socioeconomic status, as well as the trait of platform vulnerability, well-constructed studies and health policy development for these drivers should be continued.

## Figures and Tables

**Table 1:** Baseline characteristics of participants stratified by replacement driver.

Variable	Total	Paid Workers	Replacement Drivers	*p*-Value
Depressive symptoms				<0.001
No	1137 (100.00%)	1002 (87.36%)	135 (50.37%)	
Yes	278 (100.00%)	145 (12.64%)	133 (49.63%)	
Age				<0.001
Mean (SD)	48.09 (14.04)	46.21 (14.45)	56.11 (8.25)	
Education				0.647
Below high school	503 (100.00%)	404 (35.22%)	99 (36.94%)	
Above university	912 (100.00%)	743 (64.78%)	169 (63.06%)	
Household income				<0.001
Low income	624 (100.00%)	453 (39.49%)	171 (63.81%)	
High income	791 (100.00%)	694 (60.51%)	97 (36.19%)	
Smoking				<0.001
None	339 (100.00%)	299 (26.07%)	40 (14.93%)	
Ex-smoker	564 (100.00%)	465 (40.54%)	99 (36.94%)	
Current smoker	512 (100.00%)	383 (33.39%)	129 (48.13%)	
Alcohol consumption				0.004
None	221 (100.00%)	162 (14.12%)	59 (22.01%)	
Social drink	731 (100.00%)	608 (53.01%)	123 (45.9%)	
Heavy	463 (100.00%)	377 (32.87%)	86 (32.09%)	
Working hours				0.45
Short (<40)	760 (100.00%)	610 (53.18%)	150 (55.97%)	
Long (≥40)	655 (100.00%)	537 (46.82%)	118 (44.03%)	
Muscular exercise				<0.001
None	929 (100.00%)	751 (65.48%)	178 (66.42%)	
Exerciser (>1 day)	486 (100.00%)	396 (34.52%)	90 (33.58%)	
Sleeping time				<0.001
Normal	1216 (100.00%)	1022 (89.1%)	194 (72.39%)	
Abnormal (≤5/≥10)	199 (100.00%)	125 (10.9%)	74 (27.61%)	

Values are expressed using *n* (%) or mean (standard deviation). Abbreviation: SD, standard deviation.

**Table 2:** Baseline characteristics of participants stratified by depressive symptoms.

Variable	Total	No Depressive Symptoms	Depressive Symptoms	*p*-Value
Replacement driver				<0.001
No	1147 (100.00%)	1002 (88.13%)	145 (52.16%)	
Yes	268 (100.00%)	135 (11.87%)	133 (47.84%)	
Age				0.753
Mean (SD)	48.09 (14.04)	48.03 (14.23)	48.32 (13.26)	
Education				0.306
Below high school	503 (100.00%)	412 (36.24%)	91 (32.73%)	
Above university	912 (100.00%)	725 (63.76%)	187 (67.27%)	
Household income				<0.001
Low income	624 (100.00%)	473 (41.6%)	151 (54.32%)	
High income	791 (100.00%)	664 (58.4%)	127 (45.68%)	
Smoking				<0.001
None	339 (100.00%)	294 (25.86%)	45 (16.19%)	
Ex-smoker	564 (100.00%)	469 (41.25%)	95 (34.17%)	
Current smoker	512 (100.00%)	374 (32.89%)	138 (49.64%)	
Alcohol consumption				0.57
None	221 (100.00%)	181 (15.92%)	40 (14.39%)	
Social drink	731 (100.00%)	591 (51.98%)	140 (50.36%)	
Heavy	463 (100.00%)	365 (32.1%)	98 (35.25%)	
Working hours				0.435
Short (<40)	760 (100.00%)	617 (54.27%)	143 (51.44%)	
Long (≥40)	655 (100.00%)	520 (45.73%)	135 (48.56%)	
Muscular exercise				
None	929 (100.00%)	725 (63.76%)	204 (73.38%)	0.003
Exerciser (>1 day)	486 (100.00%)	412 (36.24%)	74 (26.62%)	
Sleeping time				<0.001
Normal	1216 (100.00%)	1005 (88.39%)	211 (75.9%)	
Abnormal (≤5/≥10)	199 (100.00%)	132 (11.61%)	67 (24.1%)	

Values are expressed using *n* (%) or mean (standard deviation). Abbreviation: SD, standard deviation.

**Table 3:** Multivariable logistic regression model of depressive symptoms.

Variables	Model 1	Model 2	Final Model
Replacement driver			
No	Reference (1.00)	Reference (1.00)	Reference (1.00)
Yes	9.09 (6.49–12.74)	8.59 (6.10–12.09)	7.89 (5.53–11.26)
Age	0.98 (0.96–0.99)	0.98 (0.96–0.99)	0.97 (0.96–0.99)
Education			
Below high school		Reference (1.00)	Reference (1.00)
Above university		0.94 (0.68–1.29)	0.83 (0.60–1.15)
Household income			
High		Reference (1.00)	Reference (1.00)
Low		1.34 (1.00–1.80)	1.31 (0.97–1.77)
Smoking			
None			Reference (1.00)
Ex-smoker			1.34 (0.88–2.05)
Current smoker			1.84 (1.22–2.78)
Alcohol consumption			
None			Reference (1.00)
Social drink			1.25 (0.80–1.96)
Heavy			1.36 (0.85–2.18)
Working hours			
Short (<40)			Reference (1.00)
Long (≥40)			1.18 (0.88–1.57)
Muscular exercise			
None			Reference (1.00)
Exerciser (>1 day)			1.60 (1.16–2.21)
Sleeping time			
Normal (6–9)			Reference (1.00)
Abnormal (≤5/≥10)			1.86 (1.28–2.71)

## Data Availability

Data sharing is not applicable.

## Supplementary Materials

The following supporting information can be downloaded at: https://www.mdpi.com/article/10.3390/ijerph20010575/s1, Table S1: Baseline Characteristics of Participants stratified by replacement driver work and kind of work position; Table S2: Multivariable logistic regression model of depressive symptoms among work position; Table S3: Multivariable Poisson regression model of depressive symptoms; Table S4: Multivariable logistic regression model of severe depressive symptoms (PHQ-9≥10); Table S5: Propensity score matching of Participants stratified by replacement driver; Table S6: Balanced Accuracy and area under the curve(AUC) whether using replacement driver variable or not.
