# Supplementary material for: The Association between Replacement Drivers and Depressive Symptoms
Source: Int J Environ Res Public Health. 2022 Dec 29;20(1):575. doi: 10.3390/ijerph20010575 (PMC9819967; doi:10.3390/ijerph20010575)
Supplement: Supplementary file 1 [file ijerph-20-00575-s001.zip › ijerph-2092945-supplementary.pdf]

Table S1. Baseline Characteristics of Participants stratified by replacement driver work and kind of work position

Variable	Total	Permanent Position	Temporary Position	Replacement Driver	*p*-Value
**Depressive symptom**					<0.001
No	1137 (100.00%)	587 (87.61%)	415 (87%)	135 (50.37%)	
Yes	278 (100.00%)	83 (12.39%)	62 (13%)	133 (49.63%)	
**Age**					<0.001
Mean (SD)	48.09 (14.04)	44.18 (10.77)	49.07 (18.04)	56.11 (8.25)	
**Education**					<0.001
below high school	503 (100.00%)	172 (25.67%)	232 (48.64%)	99 (36.94%)	
above university	912 (100.00%)	498 (74.33%)	245 (51.36%)	169 (63.06%)	
**Household Income**					<0.001
low income	624 (100.00%)	176 (26.27%)	277 (58.07%)	171 (63.81%)	
high income	791 (100.00%)	494 (73.73%)	200 (41.93%)	97 (36.19%)	
**Smoking**					<0.001
None	339 (100.00%)	178 (26.57%)	121 (25.37%)	40 (14.93%)	
Ex-smoker	564 (100.00%)	274 (40.9%)	191 (40.04%)	99 (36.94%)	
Current-smoker	512 (100.00%)	218 (32.54%)	165 (34.59%)	129 (48.13%)	
**Alcohol consumption**					<0.001
None	221 (100.00%)	70 (10.45%)	92 (19.29%)	59 (22.01%)	
social drink	731 (100.00%)	372 (55.52%)	236 (49.48%)	123 (45.9%)	
Heavy	463 (100.00%)	228 (34.03%)	149 (31.23%)	86 (32.09%)	
**Working Hour**					<0.001
Short (<40)	760 (100.00%)	317 (47.31%)	293 (61.43%)	150 (55.97%)	
Long (>=40)	655 (100.00%)	353 (52.69%)	184 (38.57%)	118 (44.03%)	
**Muscular exercise**					0.826
None	929 (100.00%)	443 (66.12%)	308 (64.57%)	178 (66.42%)	
exerciser(>1day)	486 (100.00%)	227 (33.88%)	169 (35.43%)	90 (33.58%)	
**Sleeping time**					<0.001
Normal	1216 (100.00%)	609 (90.9%)	413 (86.58%)	194 (72.39%)	
Abnormal	199 (100.00%)	61 (9.1%)	64 (13.42%)	74 (27.61%)	

Values are expressed by n (%) or mean (standard deviation).

Abbreviation: SD, standard deviation

Table S2. Multivariable logistic regression model of depressive symptoms among work position

Variables	Model 1	Model 2	Final Model
**work position**			
permanent worker	reference(1.00)	reference(1.00)	reference(1.00)
temporary worker	1.14 (0.80-1.62)	1.06 (0.74-1.54)	1.11 (0.76-1.61)
replacement driver	9.60 (6.63-13.92)	8.83 (6.03-12.93)	8.27 (5.56-12.30)
**Age**	0.98 (0.96-0.99)	0.98 (0.96-0.99)	0.97 (0.96-0.99)
**Education**			
below high school		reference(1.00)	reference(1.00)
above university		0.93 (0.68-1.29)	0.82 (0.59-1.14)
**Household Income**			
High income		reference(1.00)	reference(1.00)
Low income		1.32 (0.98-1.79)	1.29 (0.94-1.75)
**Smoking**			
None			reference(1.00)
Ex-smoker			1.35 (0.88-2.05)
Current-smoker			1.84 (1.22-2.77)
**Alcohol consumption**			
None			reference(1.00)
Social drink			1.26 (0.80-1.98)
Heavy			1.37 (0.85-2.20)
**Working Hour**			
Short (<40)			reference(1.00)
Long (>=40)			1.19 (0.88-1.59)
**Muscular exercise**			
None			reference(1.00)
Exerciser(>1day)			1.61 (1.16-2.22)
**Sleeping time**			
Normal (6~9)			reference(1.00)
Abnormal (<=5 ǀ >=10)			1.85 (1.27-2.70)

Table S3. Multivariable Poisson regression model of depressive symptoms

Variables	Model 1	Model 2	Final Model
**Replacement driver**			
No	reference(1.00)	reference(1.00)	reference(1.00)
Yes	4.86 (3.72-6.34)	4.66 (3.55-6.12)	4.21 (3.18-5.59)
**Age**	0.98 (0.97-0.99)	0.98 (0.97-0.99)	0.98 (0.97-0.99)
**Education**			
below high school		reference(1.00)	reference(1.00)
above university		0.96 (0.74-1.25)	0.89 (0.68-1.16)
**Household Income**			
High income		reference(1.00)	reference(1.00)
Low income		1.23 (0.96-1.58)	1.21 (0.94-1.55)
**Smoking**			
None			reference(1.00)
Ex-smoker			1.24 (0.86-1.78)
Current-smoker			1.53 (1.08-2.18)
**Alcohol consumption**			
None			reference(1.00)
Social drink			1.19 (0.83-1.72)
Heavy			1.25 (0.85-1.83)
**Working Hour**			
Short (<40)			reference(1.00)
Long (>=40)			1.38 (1.05-1.80)
**Muscular exercise**			
None			reference(1.00)
Exerciser(>1day)			1.11 (0.88-1.42)
**Sleeping time**			
Normal (6~9)			reference(1.00)
Abnormal (<=5 ǀ >=10)			1.45 (1.09-1.94)

Table S4. Multivariable logistic regression model of severe depressive symptoms (PHQ-9≥10)

Variables	Model 1	Model 2	Final Model
**Replacement driver**			
No	reference(1.00)	reference(1.00)	reference(1.00)
Yes	8.56 (5.21-14.08)	7.64 (4.61-12.67)	6.60 (3.89-11.19)
**Age**	0.98 (0.97-1.00)	0.98 (0.96-1.00)	0.98 (0.96-1.00)
**Education**			
below high school		reference(1.00)	reference(1.00)
above university		0.83 (0.51-1.36)	0.75 (0.45-1.26)
**Household Income**			
High income		reference(1.00)	reference(1.00)
Low income		1.77 (1.11-2.82)	1.70 (1.05-2.74)
**Smoking**			
None			reference(1.00)
Ex-smoker			1.43 (0.71-2.89)
Current-smoker			1.76 (0.89-3.47)
**Alcohol consumption**			
None			reference(1.00)
Social drink			0.92 (0.48-1.76)
Heavy			1.08 (0.55-2.13)
**Working Hour**			
Short (<40)			reference(1.00)
Long (>=40)			0.98 (0.62-1.55)
**Muscular exercise**			
None			reference(1.00)
Exerciser(>1day)			2.97 (1.65-5.36)
**Sleeping time**			
Normal (6~9)			reference(1.00)
Abnormal (<=5 ǀ >=10)			2.63 (1.59-4.36)

Table S5. Propensity score matching of Participants stratified by replacement driver

Variable	Total	Paid Worker	Replacement Driver	*p*-Value
**Depressive symptom**				<0.001
No	369 (100.00%)	235 (89.02%)	134 (50.76%)	
Yes	159 (100.00%)	29 (10.98%)	130 (49.24%)	
**Age**				0.727
Mean (SD)	55.88 (10.48)	55.72 (12.32)	56.04 (8.24)	
**Education**				0.788
below high school	202 (100.00%)	103 (39.02%)	99 (37.5%)	
above university	326 (100.00%)	161 (60.98%)	165 (62.5%)	
**Household Income**				>0.999
low income	335 (100.00%)	168 (63.64%)	167 (63.26%)	
high income	193 (100.00%)	96 (36.36%)	97 (36.74%)	
**Smoking**				0.632
None	77 (100.00%)	37 (14.02%)	40 (15.15%)	
Ex-smoker	190 (100.00%)	91 (34.47%)	99 (37.5%)	
Current-smoker	261 (100.00%)	136 (51.51%)	125 (47.35%)	
**Alcohol consumption**				0.856
None	119 (100.00%)	62 (23.49%)	57 (21.59%)	
Social drink	245 (100.00%)	122 (46.21%)	123 (46.59%)	
Heavy	164 (100.00%)	80 (30.3%)	84 (31.82%)	
**Working Hour**				0.793
Short (<40)	294 (100.00%)	145 (54.92%)	149 (56.44%)	
Long (>=40)	234 (100.00%)	119 (45.08%)	115 (43.56%)	
**Muscular exercise**				0.855
None	347 (100.00%)	172 (65.15%)	175 (66.29%)	
Exerciser(>1day)	181 (100.00%)	92 (34.85%)	89 (33.71%)	
**Sleeping time**				0.617
Normal	394 (100.00%)	200 (75.76%)	194 (73.48%)	
Abnormal	134 (100.00%)	64 (24.24%)	70 (26.52%)	

Table S6. Balanced Accuracy and area under the curve(AUC) whether using replacement driver variable or not

Machine Learning	*without Replacement Driver*		*with Replacement Driver*	
	Balanced Accuracy	*AUC*	*Balanced Accuracy*	*AUC*
GLM	*0.629*	*0.654*	*0.715*	*0.769*
LDA	*0.629*	*0.654*	*0.719*	*0.791*
QDA	*0.562*	*0.643*	*0.698*	*0.704*
GBM	*0.623*	*0.674*	*0.707*	*0.739*

Age, educational level, household income level, working hours, smoking status, alcohol consumption history, muscular exercise days, and sleeping time were used as covariates in the above prediction models.
